# People affected by cancer and their carers from gender and sexually diverse communities: their experiences and the role of smartphone applications

**DOI:** 10.1186/s12889-024-19144-y

**Published:** 2024-06-20

**Authors:** Natalie Winter, Anna Ugalde, Elisabeth Coyne, Karin B. Dieperink, Hannah Jongebloed, Patricia Livingston

**Affiliations:** 1 School of Nursing and Midwifery, The Centre for Quality and Patient Safety in the Institute for Health Transformation, Deakin University, Geelong, 3220 Australia; 2 School of Nursing, Griffith University, Brisbane, Australia; 3 School of Nursing, University of Southern Denmark, Odense, Denmark; 4 Family Focused Healthcare Research Center FaCe, University of Southern Denmark, Odense, Denmark; 5 Department of Oncology, Odense University Hospital, Odense, Denmark; 6 Faculty of Health, Deakin University, Geelong, 3220 Australia

**Keywords:** Neoplasm, Patient, Caregiver, Gender diverse, Sexually diverse, Digital health, LGBTQIA+, Psycho-oncology, Supportive care

## Abstract

**Background:**

People living with cancer, or carers who are from lesbian, gay, bisexual, transgender, queer, intersex or asexual (LGBTQIA+) communities experience unique information and support needs. Accessible technology-based resources providing tailored support are required to promote wellbeing, however this is a growing area of research requiring further investigation. The purpose of this study was to explore the experiences of healthcare services among people living with cancer, and their carers, who belong to sexual or gender diverse communities (LGBTQIA+), and identify how smartphone applications (apps) could support people from LGBTQIA + communities.

**Methods:**

This was a qualitative descriptive study where people living with cancer or carers from LGBTQIA + communities participated in phone interviews. Participants were recruited across Australia via social media advertisements, LGBTQIA + medical practices, and cancer advocacy groups. Participants were asked questions about their experiences, and were provided with screenshots of an existing app and asked to provide feedback on content and inclusiveness. Transcripts were coded and codes grouped together to form similar and concepts. Inductive and deductive analyses were used to create themes.

**Results:**

13 patients (mean age 56 (SD:13)), and three carers (mean age 64 (SD:19)) completed phone interviews. The majority of participants identified their gender as female (patients *n* = 9, carers 3), and their sexuality as gay or lesbian (patients *n* = 10, carers *n* = 3). Four themes were created: (1) *navigating disclosure in healthcare*, described emotional challenges surrounding disclosure; (2) *the power of positive experiences with clinicians*, described positive interactions and gaps in care from clinicians; (3) *impact of gender and sexuality on informal support*, outlined support received from informal network and gaps in support, and; (4) *opportunities to increase inclusivity in smartphone apps*, generated ideas on how apps can be tailored to meet needs identified.

**Conclusion:**

Disclosure of gender or sexuality, and interactions with clinicians had the potential to impact participants’ experience of cancer care. Gaps in informal networks pointed at how to better support LGBTQIA + communities, and identified opportunities for inclusion in an app that will be tailored and trialled for this community. Future work should focus on addressing systems-level processes in acknowledging and supporting priority groups affected by cancer.

## Background

People living with cancer often lack support related to information seeking and emotional wellbeing, while carers are also impacted by burden, family and work commitments [1–4]. Diverse patient–carer relationships may not be acknowledged across health systems designed to serve primarily heterosexual cancer patients, and resources may not address their needs [5]. Unique differences exist for people who identify as part of lesbian, gay, bisexual, transgender, queer, intersex or asexual (LGBTQIA+) communities. These can include hesitancy to disclose sexual orientation, and managing homophobic beliefs and negative behaviours from clinicians [6–8]. People in LGBTQIA + communities are also less likely to seek medical advice due to fear of discrimination and experience delayed or poor treatment options due to their sexuality [9].

Tailored interventions are needed to address disparities among LGBTQIA + patients and their carers [5]. For example, access to supportive cancer services has been identified by clinicians as a gap in the provision of care, with a particular need to support people from diverse backgrounds [10]. Despite oncologists’ willingness to meet the unmet needs of people within LGBTQIA + communities, there is evidence that clinicians lack knowledge in providing tailored care [11].

There is little literature describing how LGBTQIA + patients and carers receive support across the cancer trajectory [12]. Previous research has identified that to improve and tailor support for LBGTQIA + patients and carers, it is vital to understand what support is currently received and potential gaps in care [13]. LGBTQIA + patients are more likely to report distrust in accessing cancer services, and many same sex carers are overlooked by medical professionals [14]. Research is underway describing the experiences of survivors, carers and professionals to inform policy and service delivery [15]. However, there is a need to provide people living with cancer and carers from LBGT + communities with access to resources outside of the clinical setting, and consumer involvement is needed to develop appropriate interventions [16].

LGBQTIA + cancer patients and carers experience many of the same stressors as heterosexual patients, but their support needs are often overlooked [17]. Research has shown that patient outcomes are improved when their caregivers are supported, yet nearly half of all carers of people diagnosed with cancer report feeling increasingly isolated [18] and are impacted by significant levels of psychological distress, often greater than the patient.

Technology-based resources have the potential to support people from LGBTQIA + communities affected by cancer. Previous technology-based interventions have improved cancer patients and carer outcomes, including psychological wellbeing [19, 20] and carer burden [21]. Technology-based interventions offer a range of programs, included mindfulness [19, 22], decision aids [23], communication skills [24] and patient monitoring [20], to name a few. Previous studies have identified the importance of smartphone apps in providing carers with privacy to seek support for their own wellbeing [25], and that apps have the potential to meet carers’ needs [26]. However, it is not known whether the support needs of carers from LGBTQIA + communities are met.

This study builds upon our program of research focused on understanding the role of smartphone applications (apps) for patients and carers affected by cancer during the illness trajectory, to provide flexible and timely support [26, 27]. The trialled apps included several sections of information and resources including: cancer information, carer information, wellbeing, social network, lifestyle, notepad, contacts and hospital information. The apps were self-guided resources which could be accessed as needed. It is unknown whether these apps provide appropriate support to people from LGBTQIA + communities to navigate the cancer trajectory.

### Theoretical framework

Qualitative description aims to understand the “who, what, where of events or experiences” [28], at the same time as exploring each individual’s unique experience [29]. This methodology was chosen for this study as there is diversity in experiences among people from each LGBTQIA + community, as well as those with different types of cancer. Qualitative description provides the opportunity to understand the intersection between cancer care, gender, and sexuality. Qualitative description can be used for developing or refining interventions within healthcare as the data stay close to experiences described by patients and carers [30], which aligns with our program of research in developing smartphone apps. The study is underpinned by the Reflexive Thematic Analysis proposed by Braun and Clarke, 2024 [31].

### Aims

To explore the health care experiences of people living with cancer and carers who belong to sexual or gender diverse communities (LGBTQIA+), and identify how smartphone applications (apps) can provide potential information and support to people from LGBTQIA + communities.

## Methods

### Methodology

This is a qualitative descriptive study consisting of one-off telephone interviews. Qualitative description was chosen for the study due to the existing variety of experiences of people from each LGBTQIA + community, and in conjunction with a cancer diagnosis [29].

### Setting

People living with cancer, or their carers who identified as belonging to LGBTQIA + communities were recruited across Australia via social media ads (Meta), LGBTQIA + medical practices, and cancer advocacy groups, i.e. Breast Cancer Network of Australia, which is Australia’s leading breast cancer consumer organisation which has a comprehensive database of people diagnosed with breast cancer, and Register4, a national database that recruits people Australia-wide who volunteer their time for cancer research projects. Team members had extensive experience recruiting people living with cancer and their carers [25, 32–34].

### Participants

People living with cancer who were over the age of 18, either living with cancer and undergoing treatment, under surveillance or who were in remission, or current or past carer/support person of someone with cancer who self-identified as LGBTQIA + were invited to participate.

People who had insufficient English language skills to participate in a phone interview in English were excluded.

### Procedure and consent

Over an 18-month period recruitment flyers were distributed through paid advertisements (Meta), in waiting rooms (LGBTQIA + medical practices) and via email (members of the BCNA and Register 4 database). Flyers included a brief description of the study, eligibility criteria, and contact details for the lead researcher.

As this study was conducted in Melbourne, Australia, during COVID-19 there were limitations in recruiting through face-to-face methods, i.e. LGBTQIA + medical practices. Melbourne experienced some of the longest community restrictions, which included the shift of medical appointments to telehealth. Because of this, recruitment heavily relied on online methods.

Purposive sampling was used during recruitment. Those interested in participating followed the link provided on the recruitment material. Participants reviewed the plain language statement and provided informed consent online. Participants completed an online demographics questionnaire and entered their name, email address and phone number; participants were contacted by the project manager to arrange an interview time. When arranging an interview time, participants’ eligibility was confirmed. Participants were emailed a screenshot of an existing app and were asked to review it prior to the interview. Informed consent to participate and to audio record interviews was reconfirmed verbally at the beginning of each interview.

This study was approved by the Deakin University Human Research Ethics Committee (ID2021-007).

### Data generation

#### Demographic characteristics

Demographic characteristics were collected from all participants including, gender, sexual orientation, age, patient or carer status, length of time since diagnosis (patients), cancer type, treatment types, current or past patient/carer status, living situation, highest level of education, state of residence, length of caring (carers) and length of relationship to patient (carers).

#### Phone interviews

Semi-structured phone interviews were used to explore participants’ experiences, including support received and gaps in support. Semi-structured interviews were chosen to explore individual’s personal perceptions, beliefs and experiences of healthcare services and the potential role of smartphone apps. This approach was deemed more appropriate than focus groups due to the sensitivity of the topics. In addition, due to the ongoing threat of COVID-19 on cancer communities, it was considered inappropriate to hold face-to-face interviews with participants. Screen shots of an existing app were provided to participants to generate discussion on how apps could be adapted to meet their needs. The interview guide was developed during our earlier studies [25, 32] examining the experiences of carers in the cancer setting. Working with our consumer representative original interview questions were used to model the current interview guide (Table 1). During the study, the questions were reviewed by a peak consumer organisation prior to commencing recruitment at this site and were suitable as of modifications were requested.

**Table 1:** Semi-structured interview guide

1. Can you tell me about your experience with cancer? 2. Where have you received support from? Prompt: i) What was your experience with clinicians, friends, family and healthcare services? ii) Can you describe whether you were provided with any referrals or recommendations on where to seek support? If so, what was the outcome of the referral or recommendation? 3. Have you felt any care or supports you have received have been impacted by your identification with the LGBTQIA + community? If so, in what way? Prompt: Please describe any negative or unexpected responses or episodes of care you received. In reference to the screenshots of the smartphone app: 4. Is there anything you like or dislike about this app? Prompt: (i) How does the appearance, content and design of the app resonate with you? (ii) Is there anything that can be modified to provide greater inclusivity? 5. Are there any resources or recommendations you have for the app to ensure it meets your needs as someone with gender or sexual diversity? Prompt: From your experience [main talking point highlighted] can you describe if, and how, a smartphone app can meet your needs.	

### Thematic analysis

#### Qualitative analysis

A qualitative descriptive approach was used to analyse interview data [35]. This theory attempts to understand people’s experiences of a particular topic, using data systematically collected, coded, categorised and analysed to identify patterns and relationships from the data [31]. Interviews were audio recorded and transcribed verbatim and then read several times to gain understanding of the content. Initially transcripts were coded and codes were discussed amongst three researchers (NW, HJ, PML) for consistency. Codes were grouped together into similar and contrasting concepts [36]. Inductive thematic analysis was used to develop sub-themes and themes related to the cancer experience, need for support, and gaps in care (see Table 2). Inductive analysis was chosen for these questions as there were no preconceived assumptions made about the data as the experiences of participants are highly subjective, and no prior coding tree was used [36]. Deductive thematic analysis was used to develop the themes related to the development of the app. Deductive analysis was deemed suitable for this stage as this study is part of a program of research, and similar questions related to app development and coding of data had occurred in our previous studies [36]. Any differences in coding or development of themes and sub-themes were discussed by three authors (NW, HJ, PML) and a consensus was reached. The group size was assessed during analysis where we collected data to the point that there were no additional issues or insights identified [37], after this point, we finished recruiting [38]. In the analysis, each participant was assigned a random identification number, e.g. P1 or C1 etc. Patients were abbreviated to an identification number starting with “P”, and carers were abbreviated to an identification number starting with “C”.

**Table 2:** Themes, sub-themes, and illustrative quotes

Theme	Subtheme	Quotes
Navigating disclosure in healthcare	Anxiety surrounding disclosure to clinicians	I think what it did [others’ horror stories] was it heightened my level of anxiety for sure and discomfort. You worry’s up there, it’s like “Oh my gosh, am I going to experience this? Are they going to be all right with me? Are they going to treat me well? P1, quote 1. I guess in the back of my mind I thought, what if they were really religious or something like that? I didn’t think I would receive bad care because of it, but it’s more there’s something that was in the back of my mind that I wasn’t really overly consciously thinking of, but it really is there…But no one I did tell, there was no issues at all, and I didn’t feel it effected my care in any way P2, quote 2. I’m a health care practitioner myself, so I have some awareness of when it’s relevant to mention it to someone…[I’m] bisexual but straight passing ‘cause I’m in a relationship. with a man. In [small town] it’s some Hicksville so there’s you know attitudes there that I don’t want to be excluded or treated poorly. P9, quote 3.
	Perceived stigma when receiving medical procedures	Just say you’re having a breast examination and the person knows that you’re a lesbian. I’m worried [they’ll think] I’ll find it sexually gratifying in some way, which is not true. But I worry about that. P2, quote 4. I was worried that having a hysterectomy and then they were going to stick me in like a women’s ward, you know, because no men have hysterectomies. And then I thought if they stick me in a men’s ward and I need treatment and care for my hysterectomy, if anyone else finds out, is that also going to be a problem? But thankfully it wasn’t, I was in the men’s ward with three other gentlemen in my ward room, but yeah, it wasn’t an issue. P1, quote 5.
	Receiving positive and negative reactions	I haven’t had any discrimination whatsoever…it’s just introducing without any embarrassment saying this is my partner. P7, quote 6. [The clinicians] never bat an eyelid, never questioned. It was just like, “Yep, this is your partner. No worries.” They would talk to her like they would anyone else. Yeah. There’s just those little idiosyncrasies that some days you just want to go yeah, you get it. P3, quote 7. This is my partner, [name]. The intonation, the physical response to that, you know, sets the tone for the rest of the interview. In my case it was just bloody positive from the male specialist. P8, quote 8. Some of them it [reactions to coming out] wasn’t ideal. You have a very, truly very professional relationship rather than something a little bit warmer. P11, quote 9. I felt like we were just an anomaly… They would give me special treatment, like allow me to stay with her for those seven days she was in hospital… or they would try so hard, I mean, that’s also off putting, but try so hard to seem okay, but you knew it was really special treatment because we were queer. But I would prefer that than experiencing homophobia. C16, quote 10.
	Emotional burden in disclosing	People don’t realise how much being gay hides who and what you feel…you’ve got to come out every day [to clinicians] and you think I don’t want to have to tell this story again. P11, quote 11. In all my appointments I’d be disclosing straight up…the fact that I’m transgender now, it was opening that up quite a lot as well and having to discuss breast cancer is a lot too. It’s triggering for me, to say the least. But it is what it is. I’m not a snowflake when it comes to medical things and really, I can take it. P1, quote 12. In some ways, it [sexuality] wasn’t relevant to my chemotherapy. P2, quote 13.
The power of positive experiences with clinicians	Being treated as someone needing cancer care	My oncologist has been exceptional. She really has done a lot of research and work in understanding how hormone therapy changes things… I appreciate the fact that this [treatment] was going to be a challenge and a lot harder for her but she took it on and it was like she didn’t even blink about it and I’m impressed. P1, quote 14. After my partner passed, there was an issue with me accessing some of the superannuation and I needed to prove my relationship and the oncologist actually wrote a letter of support to the superannuation place and that enabled me to access those funds. C13, quote 15.
	Inability to relate to participants’ needs	If you can find a counsellor or a psychologist around here [rural location]…to find them and connect with that’s never really happened for me. They just live in their own world… they come from very white bred worlds and don’t understand day-to-day living here and the issues that we deal with. P4, quote 16. P - I’m a queer woman… I just felt like there was no one to talk to about the sorts of things that I was feeling. I - With the removal of both breasts and you being a queer woman, and with the information out there being very heteronormative, how did that make you feel with identifying with yourself? P I didn’t feel that I had someone to talk to about that. Yeah. For example, I was talking to a breast care nurse first, then to the social worker, and I realised, actually our whole ways of thinking were different. I was saying I was worried about having or not having it…. she said something along the lines of, “Well, it’s a hard decision,” but she meant that she thought it was hard to decide to do it because it’s such a big operation, and I felt like we were talking on two different planes there… I felt, actually, it took more bravery on my part to not have it done. P2, quote 17.
Impact of gender and sexuality on informal support	Supportive families	We were really fortunate. I know we’re concerned with tendencies towards isolation in gay communities, but we have lived in the most beautiful community people for 30 odd years and the neighbours have dubbed us the aunties. C14, quote 18. I get a great amount of support from my fiancée and her family. I have been making friends too. I have a good amount of very close friends that give me a lot of support. I live next door to my cousin, they’re the only direct family I have contact with since my transition. They are wonderfully supportive to me. P1, quote 19. I’ve got a beautiful village here. I have my mother and brother and sister-in-law who also live in the same town as well as an aunt who is like my second mother growing up…the girls’ [children’s] father, he’s still–he lives in a cabin at the back of the same property that I’m on, so he was nice and close by to come and just step in when I wasn’t able to parent and do all that sort of stuff. P3, quote 20. I’d only just moved to Melbourne a month before I found out I had cancer, so the friendships hadn’t been forged, then I had nobody…. I have a very dear friend [name] who’s another cancer survivor… and she said to me when I told her I had cancer, she said, “You call me 24/7,” and a few times I did. P5, quote 21. Not only my mum but my whole family consider gender and sexuality very different to how I see it. We’ve never been on the same page in terms of that. Yeah. I wouldn’t discuss my feelings about [cancer with them]. P2, quote 22.
	The importance of peer support	It is [exhausting the constant worry of recurrence], and I’ve got that for the rest of my life. It got so bad that in April of this year I’d been stockpiling my morphine and sleeping tablets, I took an overdose. If there had have been just somebody that was gay that had gone through my experience that I could converse with, relate to, that would have made my journey so much easier. I’m sure they’re out there, but how you find them and how you connect is another story. P5, quote 23. I would have loved a group of queers to speak to about my experience… I was desperate for that. C16, quote 24. I’m going to be a bit crass here but how do we deal with the fact that my bodily outputs are all toxic and we want to have sex. When you’re putting it in a group for breast cancer…they just say “oh just wear condoms”, that doesn’t really work and you have to get a little bit more personal. P3, quote 25. Some people are like that [preferring to read information]. Some people aren’t; I guess [peer support] would be good to have for those people. P6, quote 26.
	The void in peer support	I work half a day a week in an LGBTI team… i provide a monthly social program to support those people who can’t get out too often… [the] programs are really helpful with people, older people in general, but particularly those people who are coping with illness, whether it’s cancer or any other aged illness that you get as you age…. there’s not any specific LGBTI cancer support group. C13, quote 27. I just felt like there was no one to talk to about the sorts of things that I was feeling which I felt were perhaps a bit different from what other people might be feeling…It felt very heteronormative, the information that’s out there. P2, quote 28. I went into a few groups overseas in both Europe and in America and transgender groups and asked if there was anyone in there that had experienced breast cancer. There wasn’t. I didn’t find any, although I did find cancer patients in there and I discussed a lot of things with them just to see what they did, what they didn’t do. P1, quote 29.
Opportunities to increase inclusivity in smartphone apps	Appearance and language	Put it the flag somewhere, with the trans one as well. They [app users] might look at it and say, oh, it’s for me as well. P7, quote 30. Just taking note if you’re creating your own content, on inclusive language. P2, quote 31. If that information had been readily available, whether it’s a section in each tab for LGBTQIA+. P12, quote 32
	Supportive care content	People in the community go through a hard time and it’s sad - it’s predominantly quite a sad community, even though there’s a lot of love in there…there’s a lot of people with very fragile mental states and they need and deserve to be able to access proper information and so I think an app is a great way to reach a lot of other people. P1, quote 32. [Include] LGBTQ safe practitioners. Kind of pull the champions or allies…if there any other support networks that exist within the community that would be good. P9, quote 34. Perhaps, actually, even a register of LGBTI-friendly doctors, specialists, or something like that. I don’t know if private health have anything along those lines. P2, quote 35. Maybe [including] something about just in terms of the role of partners in the caring role… confirmation that you have cancer and partners attending those meetings. P8, quote 36.
	Filling the gap in peer support	I was really wanting to have my experience normalised, because it felt so abnormal. I would have really loved to have had that space [online peer support] to have normalised what was going on for me. C16, quote 37. Everyone is different [in response to a LGBTQIA + chat room]. Sometimes you probably do feel like you’re isolated, especially going through all the treatments, so yeah, a chat room would probably good. P7, quote 38. Basically somewhere where you can go and just talk to people who are in a similar situation with the same sort of–or a similar sort of family setup or at least the fact of understanding when you say a word straight out, when you say, “I’m a lesbian and I need this help”, then go, “Yes, I know what you mean by that”, rather than having to explain to people who have never ever thought about it, how all these things work. P3, quote 39. [Peer support] would be good because that would be accessible. I think it can do as a sense of belonging… Being amongst your own people sort of thing, where you’re not going to get somebody randomly waving a Bible at you and quoting silly things. P4, quote 40.

#### Rigor

Participants who completed a phone interview were given the opportunity to review their transcripts in a process called member checking, to ensure the credibility of findings [39]. No participants accepted this offer. During interviews, key topics brought up by participants were summarised prior to finishing the interview. No repeat interviews were conducted. Data analysis and coding of themes was led by one author, and major themes were agreed upon (NW, HJ, PL). Phone interviews were completed by two authors (NW, AU) with PhD qualifications, both working as researchers and with extensive experience in conducting interviews, and who had no previous relationship with participants.

## The analysis

### Demographic characteristics

Overall, 13 people living with cancer and three carers were interviewed. For full demographics, see Table 3. Most participants identified their gender as female (patients *n* = 9, carers 3), and their sexuality as gay or lesbian (patients *n* = 10, carers *n* = 3). People living with cancer on average were 56 years old (SD 13) and carers were 64 years old (SD 19). On average, interviews lasted 53 min (range 19–93).

**Table 3:** Demographic characteristics of participants (*N* = 16)

	Person living with cancer (*N* = 13)	Carers (*N* = 3)
Age mean (SD)	56 (13)	64 (19)
Age range	37–75	41–77
	Frequency (%)	Frequency (%)
**Gender**		
Female	9 (23)	3 (100)
Male	3 (69)	-
Non-binary	1 (8)	-
**Sex at birth**		
Female	11 (85)	3 (100)
Male	2 (15)	-
**Sexuality**		
Heterosexual	2 (15)	-
Gay or Lesbian	10 (77)	3 (100)
Bisexual	3 (23)	-
Pansexual	1 (8)	-
Queer	6 (46)	1 (33)
Asexual	1 (8)	
**Relationship status**		
Living with partner	5 (39)	1 (33)
Married	2 (15)	-
Divorced	2 (15)	-
Separated	2 (15)	-
Never married	2 (15)	1 (33)
Widowed		1 (33)
**Highest level of education**		
Completed university degree	6 (46)	2 (67)
Completed some university	3 (23)	1 (33)
Other	4 (31)	-
**State of residence**		
Victoria	7 (54)	-
New South Wales	2 (15)	1 (33)
Queensland	2 (15)	-
Tasmania	2 (15)	-
South Australia	-	2 (67)
**Geographical location**		
Metropolitan	5 (38)	1 (33)
Regional/rural	8 (62)	2 (67)
**Type of cancer patient is living with**		
Breast	9 (69)	2 (67)
Testicular	1 (8)	-
Melanoma	1 (8)	-
Prostate		-
Thyroid	1 (8)	-
Vulva	1 (8)	-
Lung	-	1 (33)
Myeloid Leukaemia	-	1 (33)
**Person living with cancer**		
**Years since diagnosis**		
1 year or less	7 (54)	-
2–10 years	3 (23)	-
10 + years	3 (23)	-
**Living situation**		
Living with people who provide me with support	7 (54)	-
Living with people but they are not involved in my care	2 (15)	-
Not living with anyone and more support would be beneficial	1 (8)	-
Not living with anyone and not needing support	3 (23)	-
**Carers**		
**Length of caring period**		
1–2 years	-	2 (67)
3–5 years	-	1 (33)
**Relationship to person with cancer**		
Spouse/de facto	-	2 (67)
Friend	-	1 (33)
**Number of years carers have known the person living with cancer**		
10–19 years	-	2 (67)
30 + years	-	1 (33)
**Currently providing care**		
Yes	-	2 (67)
**Living situation**		
I don’t live with others	-	2 (67)
I live with the person receiving care	-	1 (33)

### Themes

Four themes were created; (1) Navigating disclosure in healthcare (2) The power of positive experiences with clinicians; (3) Impact of gender and sexuality on informal support, and; (4) Opportunities to increase inclusivity in smartphone apps.

#### Theme one: Navigating disclosure in healthcare

Theme one describes participants’ experiences when deciding whether to disclose their gender or sexuality, and the potential consequences of disclosure. Three subthemes were developed: (i) perceived stigma when receiving medical procedures, (ii) anxiety surrounding disclosure to clinicians, (iii) receiving positive and negative reactions, (iv) emotional burden in disclosing.

##### Sub-theme one - Anxiety surrounding disclosure to clinicians

Participants reported anxiety in having to disclose their gender or sexuality to their clinicians. Anxieties stemmed from hearing of others’ negative experiences when disclosure to clinicians (P1, quote 1), judgement from people with religious faith (P2, quote 2), and potential narrow mindedness of people living in small communities (P9, quote 3). One of these participants had earlier described having a positive experience coming out. Despite this, she still felt hesitation when disclosing her sexuality (P2, quote 2).

##### Sub-theme two - Perceived stigma when receiving medical procedures

Some participants described that they were concerned with experiencing stigma when receiving medical procedures if they were to disclose their gender or sexuality. One participant felt stigma could come from clinicians who may assume they find intimate medical procedures as “sexually gratifying” (P2, quote 4). One participant who was transgender felt he may experience stigma from other patients overhearing discussions with his clinicians about his recovery, and he was conscious of others feeling discomfort in his presence (P1, quote 5).

##### Sub-theme three - Receiving positive and negative reactions

Eleven participants described their experience in disclosing their sexuality to clinicians. Five participants reported that their disclosure to clinicians was natural however, required a direct approach to communication (for example, P7, quote 6; P3, quote 7; P8, quote 8). However, two participants felt that disclosure resulted in either less empathetic care (P11-Patient; Quote 9), or “really special treatment” (C16, quote 10).

##### Sub-theme four - Emotional burden in disclosing

Two participants described the mental burden of having to disclose their personal information where the same burden did not exist for people who were heterosexual or cisgender (P11, quote 11, P1, quote 12). Alternatively, one participant stated she felt no burden or inclination to disclose her sexuality as it was not necessary for the care she was receiving (P2, quote 13).

#### Theme two: The power of positive experiences with clinicians

In theme two, we describe how experiences with clinicians following disclosure impacted on the care received by participants. We outline two contrasting sub-themes, the first is being treated as someone needing cancer care, and the second is the inability to relate to patients’ or carers’ needs.

##### Sub-theme one - Being treated as someone needing cancer care

Participants highlighted the need to primarily be seen by clinicians in a way that was integral to their care. Positive experiences with clinicians led to an overall positive experiences of cancer care. For one participant who was transgender it was important that his gender was considered when making decisions about treatment, in this way his transition was acknowledged and supported, rather than him being provided with standard treatment which would impact his transition (P1, quote 14). Alternatively, participants described the need to be seen and supported with no regard given to their sexuality as it was not relevant to treatment (C13, quote 15).

##### Sub-theme two - Inability to relate to patients’ or carers’ needs

Contrastingly, other participants felt there were noticeable gaps in their care, in particular, emotional support, as clinicians were not about to relate to or fully understand their circumstances (P4, quote 16; P2, quote 17).

#### Theme three: Impact of gender and sexuality on informal support

The importance of informal support was developed in theme three. While supportive families were important for participants’ wellbeing, the concept of needing support as a person affected by cancer *and* from LGBTQIA + communities was largely discussed. Three sub-themes were created: supportive families, the importance of peer support, and the void in peer support.

##### Sub-theme one–Supportive families

Participants noted that acceptance of their gender or sexuality from their family and community had an impact on their emotional wellbeing, and opportunities to receive practical support. In most cases, participants spoke of living in accepting families and communities (for example, C14, quote 18; P1, quote 19; P3, quote 20; P5, quote 21). Less frequently, participants described that their family did not accept their sexuality and as a result, participants did not receive support from them (P2, quote 22).

##### Sub-theme two - The importance of peer support

Both people living with cancer and carers spoke of the need for peer support from people living with cancer who were also from LGBTQIA + communities. This spoke to a larger issue of needing adequate emotional support (P5, quote 23; C16, quote 24), or practical advice specific to their situation (P3, quote 25). One patient felt they would not use peer support themselves, but noted its importance for others (P6, quote 26).

##### Sub-theme three - The void in peer support

There was a noticeable absence of peer support for people affected by cancer and who were from LGBTQIA + communities (C13, quote 27; P2, quote 28). At times, this meant that participants had to seek support internationally, however, this still did not meet their needs (P1, quote 29).

#### Theme four: Opportunities to increase inclusivity in smartphone apps

Ideas generated in this theme surrounded how to improve inclusivity in cancer related smartphone apps for people from LGBTQIA + communities. Three sub-themes were created: appearance and language, supportive care content, and filling the gap in peer support.

##### Sub-theme one - Appearance and language

Visual cues and language were noted as having the potential to promote inclusivity in smartphone apps. Minor but important suggestions were made, such as the rainbow and trans flag (P7, quote 30), gender neutral inclusive language (P2, quote 31), and standalone spaces for LGBTQIA + information and support (P12, quote 32).

##### Sub-theme two - Supportive care content

Participants felt that a smartphone app was an important resource in being able to provide support to people from LGBTQIA + communities affected by cancer (P1, quote 33). Apps have the potential to link people in with “safe practitioners” who were allies of people from LGBTQIA + communities, by having recommendations for inclusive clinicians (P9, quote 34; P2, quote 35). Additionally, one participant highlighted the importance of recognising the role of the family support person in the cancer journey (P8, quote 36).

##### Sub-theme three – filling the gap in peer support

Access to peer support was a recurring method of how to facilitate connection with others. Participants described that peer support could be embedded into apps through chat rooms which had dedicated space for LGBTQIA + groups, facilitating their access to emotional and practical support (C16, quote 37; P7, quote 38; P3, quote 39; P4, quote 40).

## Discussion

People living with cancer and carers who are from LGBTQIA + communities described the decision to disclosure their gender or sexuality could have perceived positive and negative impacts on their care and wellbeing. Both people living with cancer and carers reported the need to feel understood by clinicians and informal support networks, and this influenced their experience of cancer care. Both people living with cancer and carers suggested that inclusivity can be supported within apps by incorporating visual cues, language and peer support such as chat rooms.

This paper adds evidence to the literature regarding the need for inclusive and safe healthcare environments for people from LGBTQIA + communities, encompassing cancer care and peer support. Additionally, findings provide insight for clinicians in how to better support people from LGBTQIA + communities, including non-judgemental language, acceptance of carers regardless of their gender, sexuality or relationship to the patient, and proactively providing emotional support to patients and carers.

Participants reported disclosure could impact on their ability to receive medical care, psychosocial support and to feel seen by clinicians. Similar to previous studies, hesitancy to disclose gender or sexuality occurred due to fear of poor medical care and judgement either from clinicians or family [6, 9, 40]. As awareness and acceptance of LGBTQIA + communities grow, there is a need to provide safe spaces for people to disclose their gender or sexuality in healthcare settings [7] and to be met with culturally competent care [8]. Clearer identification of people from LGBTQIA + communities in the cancer setting can highlight gaps in care and allow for appropriate allocation of supportive resources [41] and can facilitate improvements in health outcomes [42]. There is a need to implement these strategies within apps and future interventions to provide people with safety while awaiting cultural shifts in clinical practice. In our study, the concept of promoting a safe space within apps included displaying the rainbow and transgender flag and having dedicated LGBTQIA + sections. These findings align with previous studies that have described the same methods for promoting inclusivity within clinical settings [7]. Importantly, participants noted linking to “safe” practitioners within apps would assist them in knowing where to access indiscriminatory healthcare. Patient, carer, clinician, and public involvement in the development of apps, such as the use of co-design frameworks, can promote inclusivity of all priority groups in the community [43, 44]. Use of co-design may also provide clinicians with first-hand information about the needs of each priority group, and how their needs can be met within clinical practice.

Previous studies have shown that people from LGBTQIA + communities are more likely to rely on support from friends compared to partners or family members [45]. In the current study, participants described support from family and community, however, the most prominent need for support was from peer connections. Peer connection allows people to find benefit from the giving and receiving of support [46]. Peer support has a positive impact of patients’ wellbeing across a variety of cancer types [47], and peer support for carers has been testing using social media groups [48] and peer developed programs [49] showing promising results. Additional research is needed for carer peer support to strengthen these findings. Peer support among LGBTQIA + groups has strong evidence in supporting mental wellbeing, particularly within the transgender community [50, 51]. However, there are few peer support programs for people affected by cancer and who are from LGBTQIA + communities [40], and a systematic review in 2021 highlighted that these gaps also exist in the provision of psychosocial care [41]. Participants described that peer support could be delivered in apps through the inclusion of chat rooms. Peer connections via e-health technologies have similarly been identified as an area of need among transgender people [52] and men from sexual minorities [53]. Peer support is available widely online, however participants described that including peer support forums within apps, such as chat rooms, could promote inclusivity and bridge the gap between LGBTQIA + peer support and cancer peer support.

This study is limited by the small sample size and homogeneity of carers, with most being cisgender females or cisgender males and highly educated; this is consistent with other studies in the cancer setting [54, 55]. The majority of participants were diagnosed with breast cancer, which is consistent with previous research and demonstrates that understanding the role of diversity among different cancer types remains a challenge even in studies focusing on priority groups. A strength of this study was the range of people who participated nationally and from rural and regional areas. These demographics provide us with a greater understanding of people’s experiences with cancer across Australia, and the potential of an app to meet the needs of diverse groups. However, larger studies are needed to validate these findings.

As participants were aware that this study was part of a wider research project focused on the topic of smartphone apps, it is possible that this impacted on who participated due to interest in the topic. While this may have resulted in a more homogenous sample, we are confident that key experiences described are similar across different cohorts as no new concepts were discussed, and our findings are similar to other literature describing the experiences of people from LGBTQIA + communities [50, 51]. Similarly, the initial concept and design of the app had been developed in previous studies and participants were provided with screenshots of the existing app. This may have influenced participants’ generation of ideas regarding app content and design.

## Conclusion

Disclosure of gender or sexuality was a personal decision among people living with cancer and carers from LGBTQIA + communities, due to uncertainty of the impact of disclosure on care provided. There is a need for people from LGBTQIA + communities to be seen and understood by clinicians in a way that supports their care needs. Modification in the visuals, language and structure of smartphone apps can improve inclusivity. Resources such as peer support have the potential to provide informal support and should be included in the app. Additional research is needed to trial an app which incorporates visuals, language, structural changes and peer support for people living with cancer and carers from LGBTQIA + communities. Priorities for future work should also focus on addressing systems-level processes in acknowledging and supporting priority groups affected by cancer and their carers.

## Data Availability

The datasets used and/or analysed during the current study are available from the corresponding author on reasonable request.

## Abbreviations

LGBTQIA+: Lesbian, gay, bisexual, transgender, queer, intersex or asexual
